# Deletion of Chromosomes 13q and 14q Is a Common Feature of Tumors with BRCA2 Mutations

**DOI:** 10.1371/journal.pone.0052079

**Published:** 2012-12-21

**Authors:** Audrey Rouault, Guillaume Banneau, Gaëtan MacGrogan, Natalie Jones, Nabila Elarouci, Emmanuelle Barouk-Simonet, Laurence Venat, Isabelle Coupier, Eric Letouzé, Aurélien de Reyniès, Françoise Bonnet, Richard Iggo, Nicolas Sévenet, Michel Longy

**Affiliations:** 1 French National Institute of Health and Medical Research (INSERM) Unit 916, University of Bordeaux, Bergonié Cancer Institute, Bordeaux, France; 2 Pathology Department, Bergonié Cancer Institute, Bordeaux, France; 3 Cartes d’Identité des Tumeurs (CIT) Program, Ligue Nationale Contre Le Cancer, Paris, France; 4 Cancer Genetics Unit, Bergonié Cancer Institute, Bordeaux, France; 5 Department of Medical Oncology, Dupuytren University Hospital, Limoges, France; 6 Cancer Genetics Unit, Val d’Aurelle Regional Cancer Centre, Montpellier, France; Cedars-Sinai Medical Center, United States of America

## Abstract

**Introduction:**

Germline *BRCA1* or *BRCA2* mutations account for 20–30% of familial clustering of breast cancer. The main indication for *BRCA2* screening is currently the family history but the yield of mutations identified in patients selected this way is low.

**Methods:**

To develop more efficient approaches to screening we have compared the gene expression and genomic profiles of *BRCA2*-mutant breast tumors with those of breast tumors lacking *BRCA1* or *BRCA2* mutations.

**Results:**

We identified a group of 66 genes showing differential expression in our training set of 7 *BRCA2*-mutant tumors and in an independent validation set of 19 *BRCA2*-mutant tumors. The differentially expressed genes include a prominent cluster of genes from chromosomes 13 and 14 whose expression is reduced. Gene set enrichment analysis confirmed that genes in specific bands on 13q and 14q showed significantly reduced expression, suggesting that the affected bands may be preferentially deleted in *BRCA2*-mutant tumors. Genomic profiling showed that the *BRCA2*-mutant tumors indeed harbor deletions on chromosomes 13q and 14q. To exploit this information we have created a simple fluorescence in situ hybridization (FISH) test and shown that it detects tumors with deletions on chromosomes 13q and 14q.

**Conclusion:**

Together with previous reports, this establishes that deletions on chromosomes 13q and 14q are a hallmark of *BRCA2*-mutant tumors. We propose that FISH to detect these deletions would be an efficient and cost-effective first screening step to identify potential *BRCA2*-mutation carriers among breast cancer patients without a family history of breast cancer.

## Introduction

Germline mutations in pathways critical for maintenance of genomic integrity confer an increased risk of developing breast cancer [1]. Inherited mutations in two genes, breast cancer 1 (*BRCA1*) and *BRCA2*, are associated with a particularly striking increase in breast cancer risk [2]. Consistent with the Knudson two-hit model, both alleles of *BRCA1* and *BRCA2* are inactivated in tumors, indicating that the genes behave like classic tumor suppressor genes [3]. Their gene products are implicated in the repair of DNA double-strand breaks [4]: BRCA1 is required for recruitment of repair proteins to sites of breakage [5], whereas BRCA2 nucleates RAD51 filament assembly on single-stranded DNA exposed by resection from the break [6]. Loss of these functions leads to genomic instability [7].

The criteria used to select patients for *BRCA2* screening are essentially based on the family history. Unfortunately, this approach is wasteful of resources because relatively few familial clusters are caused by germline *BRCA2* mutations [8]. This approach also overlooks patients with no overt family history of breast or ovarian cancer who may nevertheless have *BRCA2* mutations. Despite numerous efforts, no specific clinical or pathological features have been identified that permit easy identification of *BRCA2*-associated tumors.

The role BRCA2 plays in repair of double strand breaks by homologous recombination might be expected to give a characteristic pattern of genomic instability but no genomic features have yet been described that can be used to identify these tumors. Gene expression profiling typically places the tumors in the luminal B, high proliferation, estrogen receptor (ER) positive group of the Stanford classification but this is not specific enough to be useful clinically to identify tumors with *BRCA2* mutations [9].

In this study, we have used gene expression and genomic data to identify specific molecular features that distinguish tumors with *BRCA2* mutations from tumors with other breast cancer predisposition mutations. Based on these results we have developed a fluorescent *in-situ* hybridization (FISH) test that can be used to screen for tumors with an increased risk of containing *BRCA2* mutations.

## Methods

### Patients and Samples

All samples were from the Bergonie Cancer Institute, Bordeaux, except for sample 144 from the Val d’Aurelle Regional Cancer Center, Montpellier; samples 146 and 148 from the Dupuytren Hospital, Limoges; and the BRCA2 tumors in the validation set from the Curie Institute. The microarray data for the validation set were generously provided by the Translational Research Unit at the Curie Institute, Paris. The control group contained *BRCAX* tumors, defined as tumors lacking known *BRCA1/2* mutations from families with either i) at least three breast cancer-affected first or second-degree relatives; or ii) breast cancer before age 42 or ovarian cancer in two first-degree relatives or two second-degree relatives via a male. All patients agreed to the use of their samples for research purposes, in compliance with the French law on tumor banks (law number 2004-800, French Public Health Code articles L. 1243-4 and R. 1243-61) under authorisation number AC-2008-812, which was approved by the Comité de Protection des Personnes. The *BRCA1* and *BRCA2* mutation search was made after patients gave signed informed consent in the context of a medical genetic diagnosis of suspected breast cancer predisposition, in compliance with the French law on genetic testing (law number 94-654).

### Tumor and Mutation Characterization

Clinical, pathological and genetic data for each case are listed in Table 1. Immunohistochemistry for ER, progesterone receptor (PR) and HER2 (ERBB2) were performed as previously described [10]. HER2 expression was scored according to the Herceptest system. ER and PR were scored by multiplying the percentage of positive cells by the intensity (score 0–20: −; score 21–100: +; score 101–200: ++; score 201–300: +++). Screening for germline mutations was performed on leucocyte DNA as previously described [10].

**Table 1:** Characteristics of patients and tumors.

ID	Tumor set	BRCA status	Sex	Age at surgery (year)	Tumor size (mm)	Tumor cells (%)	Histologic grade	ER	PR	ERBB2
52	Training	BRCA2	F	35	17	92	3	++	−	−
86	Training	BRCA2	F	46	16	90	3	+++	+	−
106	Training	BRCA2	F	57	22	85	3	+++	+	−
133	Training	BRCA2	F	40	15	75	2	+	+	−
144	Training	BRCA2	F	40	12	55	2	++	−	+
146	Training	BRCA2	F	64	25	80	3	−	−	+
148	Training	BRCA2	F	62	25	90	3	++	−	++
8	Training	BRCAX	F	51	18	90	3	−	−	−
9	Training	BRCAX	F	51	25	95	3	++	++	−
11	Training	BRCAX	F	56	40	78	2	++	+++	−
14	Training	BRCAX	F	45	12	90	2	nd	+++	−
16	Training	BRCAX	F	50	27	95	3	+++	+++	−
22	Training	BRCAX	F	64	18	90	2	+++	+	−
24	Training	BRCAX	F	35	12	70	1	++	−	−
25	Training	BRCAX	F	37	12	92	2	++	+	−
33	Training	BRCAX	F	42	35	73	1	++	−	−
37	Training	BRCAX	F	45	20	92	2	+++	+++	−
38	Training	BRCAX	F	64	13	90	3	+++	−	−
40	Training	BRCAX	F	41	12	95	2	+	++	−
41	Training	BRCAX	F	38	21	92	3	++	+	−
46	Training	BRCAX	F	60	38	90	2	−	−	−
66	Training	BRCAX	F	73	12	90	2	+++	+++	−
75	Training	BRCAX	F	58	14	80	2	−	−	+++
79	Training	BRCAX	F	42	11	90	3	++	++	−
81	Training	BRCAX	F	46	28	80	2	+	++	−
82	Training	BRCAX	F	50	9	85	1	++	++	−
84	Training	BRCAX	F	47	27	92	3	++	+	+
85	Training	BRCAX	F	64	15	90	1	−	+++	−
93	Training	BRCAX	F	44	18	85	2	+++	+++	−
107	Training	BRCAX	F	69	40	80	3	+++	+	−
111	Training	BRCAX	F	73	15	80	1	+++	+	−
3	Validation	BRCAX	F	36	18	95	3	−	+	+++
15	Validation	BRCAX	F	42	15	95	3	−	−	−
17	Validation	BRCAX	F	76	3	95	1	+++	+	−
30	Validation	BRCAX	F	51	nd	95	3	+++	−	nd
48	Validation	BRCAX	F	54	20	90	1	++	++	−
49	Validation	BRCAX	F	49	35	66	2	+++	++	−
65	Validation	BRCAX	F	46	37	95	3	−	−	−
71	Validation	BRCAX	F	43	21	73	2	++	+++	+++
83	Validation	BRCAX	F	50	18	50	2	++	−	−
89	Validation	BRCAX	F	30	30	82	nd	++	+++	−
96	Validation	BRCAX	F	41	25	85	3	−	−	+++
99	Validation	BRCAX	M	63	21	90	1	+++	++	−
43	Genomic	BRCA2	F	38	12	90	2	++	−	−
149	Genomic	BRCA2	F	76	70	60	2	+++	−	−

Footnote. Tumor set: Training set, tumors used to create the gene expression signature; Validation set, BRCAX tumors from Bergonie Cancer Institute; Genomic set, tumors only used for CGH and SNP analysis. nd, not determined. There was no statistically significant difference (p>0.05, Fisher test) between the BRCA2 and BRCAX groups for the following comparisons: age at surgery<vs ≥49 years (median age); tumor size<vs ≥18 mm (median tumor size); tumor cell content<vs ≥90% (median tumor cell content);+++vs other ER status; − vs other PR status; − vs other ERBB2 status.

### Gene Expression and Genomic Chip Hybridization

RNA was extracted from the tumors as described [10] and hybridized to Affymetrix U133 Plus 2.0 genechip microarrays by the Genopole Alsace-Lorraine genomics platform, except for the validation set which was hybridized by the Curie Institute genomics platform. DNA was extracted from the tumors and hybridized to Integrachip V7 bacterial artificial chromosome (BAC) arrays as described [10]. SNP array profiling was performed on Illumina Human610-Quad v1.0 BeadChips (Illumina, Inc., San Diego, CA) by Integragen (Evry, France). The gene expression and genomic data are available in Array Express under accession numbers E-TABM-854, E-MEXP-3688, E-MEXP-3690 and in GEO under accession number GSE39710.

### Data Processing and Statistical Analyses

Given the rarity of the tumors, it was not possible to avoid processing the tumors in batches; the hybridization dates for the Affymetrix chips are given in the CEL files. The 12 *BRCAX* controls for the validation set were chosen because they showed the smallest batch effect relative to the Curie Institute tumors. The 12 *BRCAX* tumors in the validation set were separate from the 24 *BRCAX* tumors in the training set. The gene expression data were normalized with the RMA algorithm in R version 2.13.1 [11]–[13]. To eliminate redundant genes sharing a gene symbol, the most variable probeset was selected based on the standard deviation across the entire dataset. Differentially expressed genes were identified by moderated t-test in limma [14] (an R script for the expression analysis is available on request). The 66 BRCA2 gene signature genes were combined to make a BRCA2 score by summing the mean-centered expression values weighted by the t values from limma. Gene Set Enrichment Analysis (GSEA) was performed with Broad Institute java software [15], [16]: the expression dataset was ranked by t-statistic in limma, then enrichment was scored by GSEA for chromosome bands using the MSigDB positional gene sets [15], [16]. Centroid-linkage hierarchical clustering was performed in Cluster 3.0 and visualized in TreeView [17]. Array CGH data was normalized with CAPweb software [18] and genomic alterations were visualized with VAMP software using the same thresholds as previously described [10]. SNP data were normalized with Illumina Genome Studio Software v2010.1 using Genotyping module (v1.6.3) and Illumina Genome Viewer module (v1.6.1) to obtain the B Allele Frequency (BAF) for each SNP.

### Fluorescence *In Situ* Hybridization

To detect deletions on chromosomes 13 and 14, FISH was performed with four BAC probes supplied by BlueGnome (Cambridge, UK). Two clones labeled with SpectrumGreen were used to detect the pericentromeric regions of chromosomes 13 and 14: RP11-408E5 on 13q12.11 (hg19 chr13∶19700993–19850551); and RP11-98N22 on 14q11.2 (hg19 chr14∶20500968–20660726). Two clones labeled with SpectrumOrange (giving red spots in the figures) were used to detect the deletions on chromosomes 13 and 14: RP11-71C5 on 13q14.11 (hg19 chr13∶44921196–45086777) and RP11-242P2 on 14q31.1 (hg19 chr14∶80030106–80193689). Nuclei obtained by touch imprints were fixed in 3∶1 methanol: acetic acid, washed and dried. The BAC probes were mixed, 5 µl of hybridization mix was added per slide, and a coverslip was glued in place to create a hybridization chamber. The sections were denatured at 75°C for 5 minutes and hybridized at 37°C overnight. Stringent washes were performed at 65°C for 10 minutes, then the sections were dehydrated in ethanol and mounted. Images were acquired with a Zeiss Axio Imager Z2 microscope (Gottingen, Germany). The number of red and green spots per nucleus was scored in morphologically intact and non-overlapping nuclei. Deletions were reported when ≥50% of nuclei with the modal number of green spots contained fewer red spots or when they contained single green and red spots.

## Results

### Identification of Genes Differentially Expressed in *BRCA2*-mutant Tumors

**Figure 1:**
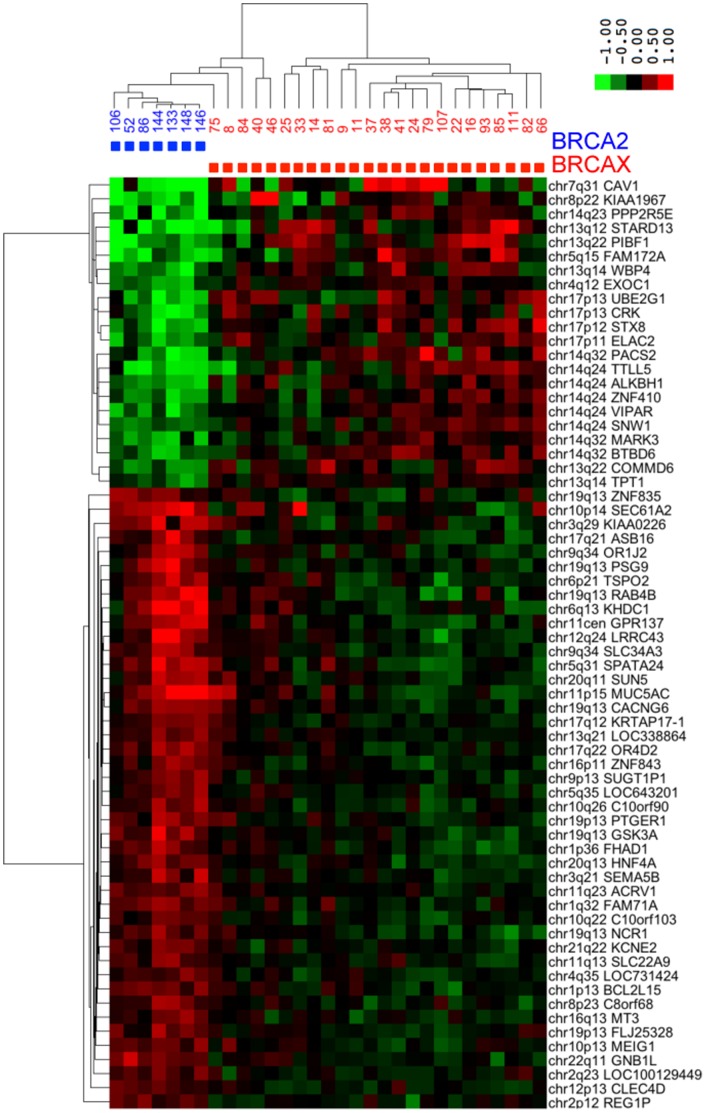
Unsupervised hierarchical clustering of the 66 *BRCA2* signature genes in the training set. There are seven *BRCA2-*mutant tumors and 24 *BRCAX* tumors (tumors from patients lacking known BRCA1/2 mutations but with a familial history of breast cancer). The upper left quadrant contains many genes on 13q and 14q that show reduced expression in *BRCA2* tumors.

To gain insight into the biology of *BRCA2*-mutant breast tumors, we performed a supervised analysis looking for genes differentially expressed in *BRCA2*-mutant and control tumors. All of the tumors came from patients with a familial clustering of breast cancer potentially caused by germline mutation of a breast cancer predisposition gene. The *BRCA2*-mutant group included 7 tumors from patients with known germline *BRCA2* mutations. The control group (“*BRCAX*”) contained 24 patients without mutations in *BRCA1* or *BRCA2* identifiable by conventional screening. RNA from these 31 tumors was tested on Affymetrix gene expression chips. Sixty-six genes were differentially expressed in the *BRCA2* and *BRCAX* groups at a false discovery rate <0.01 after Benjamini Hochberg correction for multiple testing (Table 2). Hierarchical clustering confirmed, as expected, that the differentially expressed genes cleanly split the tumors into two groups (Figure 1). The *BRCA2* group in the heatmap contains five *BRCAX* tumors that may represent tumors whose *BRCA2* mutations were missed by screening or tumors that phenocopy *BRCA2*.

**Table 2:** *BRCA2* signature genes.

Affymetrix ID	Gene Symbol	Gene Description	Band	t	p
222127_s_at	EXOC1	exocyst complex component 1	4q12	−7.05	0.0011
223564_s_at	GNB1L	G protein beta polypeptide 1-like	22q11	6.85	0.0011
632_at	GSK3A	glycogen synthase kinase 3 alpha	19q13	6.42	0.0025
1555377_at	OR4D2	olfactory receptor, family 4, subfamily D, member 2	17q22	6.13	0.0030
208429_x_at	HNF4A	hepatocyte nuclear factor 4, alpha	20q13	6.12	0.0030
207973_x_at	ACRV1	acrosomal vesicle protein 1	11q23	6.21	0.0030
218431_at	C14orf133	VPS33B interacting protein	14q24	−6.01	0.0034
1552510_at	SLC34A3	solute carrier family 34 (sodium phosphate), member 3	9q34	5.9	0.0041
204690_at	STX8	syntaxin 8	17p12	−5.84	0.0044
227630_at	PPP2R5E	protein phosphatase 2, regulatory subunit B′, epsilon	14q23	−5.7	0.0047
205621_at	ALKBH1	alkB, alkylation repair homolog 1 (E. coli)	14q24	−5.69	0.0047
202569_s_at	MARK3	MAP/microtubule affinity-regulating kinase 3	14q32	−5.74	0.0047
216520_s_at	TPT1	tumor protein, translationally-controlled 1	13q14	−5.71	0.0047
230055_at	KHDC1	KH homology domain containing 1	6q13	5.6	0.0048
221966_at	GPR137	G protein-coupled receptor 137	11cen	5.62	0.0048
207733_x_at	PSG9	pregnancy specific beta-1-glycoprotein 9	19q13	5.59	0.0048
1555614_at	SUGT1P1	suppressor of G2 allele of SKP1 (S. cerevisiae) pseudogene 1	9p13	5.57	0.0048
1552772_at	CLEC4D	C-type lectin domain family 4, member D	12p13	5.57	0.0048
203598_s_at	WBP4	WW domain binding protein 4 (formin binding protein 21)	13q14	−5.51	0.0048
1563639_a_at	FHAD1	forkhead-associated (FHA) phosphopeptide binding domain 1	1p36	5.54	0.0048
234680_at	KRTAP17-1	keratin associated protein 17-1	17q12	5.52	0.0048
1562657_a_at	C10orf90	chromosome 10 open reading frame 90	10q26	5.45	0.0055
236979_at	BCL2L15	BCL2-like 15	1p13	5.39	0.0061
221095_s_at	KCNE2	potassium voltage-gated channel, Isk-related family, member 2	21q22	5.4	0.0061
213239_at	PIBF1	progesterone immunomodulatory binding factor 1	13q22	−5.36	0.0063
1567257_at	OR1J2	olfactory receptor, family 1, subfamily J, member 2	9q34	5.34	0.0064
225389_at	BTBD6	BTB (POZ) domain containing 6	14q32	−5.31	0.0066
207778_at	REG1P	regenerating islet-derived 1 pseudogene	2p12	5.3	0.0066
226005_at	UBE2G1	ubiquitin-conjugating enzyme E2G 1 (UBC7 homolog, yeast)	17p13	−5.25	0.0070
215424_s_at	SNW1	SNW domain containing 1	14q24	−5.23	0.0070
1564112_at	FAM71A	Family with sequence similarity 71, member A	1q32	5.25	0.0070
237980_at	LINC00347	hypothetical LOC338864	13q21	5.24	0.0070
213103_at	STARD13	StAR-related lipid transfer (START) domain containing 13	13q12	−5.18	0.0071
237257_at	RAB4B	RAB4B, member RAS oncogene family	19q13	5.19	0.0071
201767_s_at	ELAC2	elaC homolog 2 (E. coli)	17p11	−5.2	0.0071
209944_at	ZNF410	zinc finger protein 410	14q24	−5.16	0.0071
1558641_at	SPATA24	spermatogenesis associated 24	5q31	5.2	0.0071
212735_at	KIAA0226	Beclin-1 associated RUN domain containing protein	3q29	5.17	0.0071
215449_at	TSPO2	translocator protein 2	6p21	5.15	0.0071
1553253_at	ASB16	ankyrin repeat and SOCS box-containing 16	17q21	5.14	0.0071
231625_at	SLC22A9	solute carrier family 22 member 9	11q13	5.2	0.0071
225312_at	COMMD6	COMM domain containing 6	13q22	−5.12	0.0074
217187_at	MUC5AC	mucin 5AC, oligomeric mucus	11p15	5.1	0.0077
1553728_at	LRRC43	leucine rich repeat containing 43	12q24	5.07	0.0079
1552863_a_at	CACNG6	calcium channel, voltage-dependent, gamma subunit 6	19q13	5.07	0.0079
217095_x_at	NCR1	natural cytotoxicity triggering receptor 1	19q13	5.06	0.0079
223610_at	SEMA5B	semaphorin 5b	3q21	5.06	0.0079
203065_s_at	CAV1	caveolin 1, caveolae protein, 22 kDa	7q31	−5.03	0.0080
202226_s_at	CRK	v-crk sarcoma virus CT10 oncogene homolog (avian)	17p13	−5.04	0.0080
235416_at	LOC643201	centrosomal protein 192 kDa pseudogene	5q35	5.03	0.0080
1557827_at	C10orf103	chromosome 10 open reading frame 103	10q22	5.03	0.0080
225187_at	KIAA1967	DBC1 deleted in breast cancer 1	8p22	−4.98	0.0082
212936_at	FAM172A	family with sequence similarity 172, member A	5q15	−4.99	0.0082
215898_at	TTLL5	tubulin tyrosine ligase-like family, member 5	14q24	−4.98	0.0082
212778_at	PACS2	phosphofurin acidic cluster sorting protein 2	14q32	−5	0.0082
1562914_a_at	FLJ25328	hypothetical LOC148231	19p13	5	0.0082
215826_x_at	ZNF835	zinc finger protein 835	19q13	4.97	0.0084
238158_at	MEIG1	meiosis expressed gene 1 homolog (mouse)	10p13	4.97	0.0084
219499_at	SEC61A2	Sec61 alpha 2 subunit (S. cerevisiae)	10p14	4.94	0.0087
207650_x_at	PTGER1	prostaglandin E receptor 1 (subtype EP1), 42 kDa	19p13	4.94	0.0087
237188_x_at	SUN5	Sad1 and UNC84 domain containing 5	20q11	4.92	0.0091
1557679_at	C8orf68	chromosome 8 open reading frame 68	8p23	4.91	0.0092
224256_at	LOC100129449	PRO2055	2q23	4.89	0.0095
1564362_x_at	ZNF843	zinc finger protein 843	16p11	4.88	0.0097
205970_at	MT3	metallothionein 3	16q13	4.87	0.0098
1569095_at	LOC731424	hypothetical LOC731424	4q35	4.87	0.0098

Footnote. t: moderated t-statistic for 66 genes that best discriminate between *BRCA2* and *BRCAX* tumors. p: p-value after Benjamini Hochberg correction (all genes had an unadjusted p-value <0.0001).

### Validation of a Putative *BRCA2* Signature

**Figure 2:**
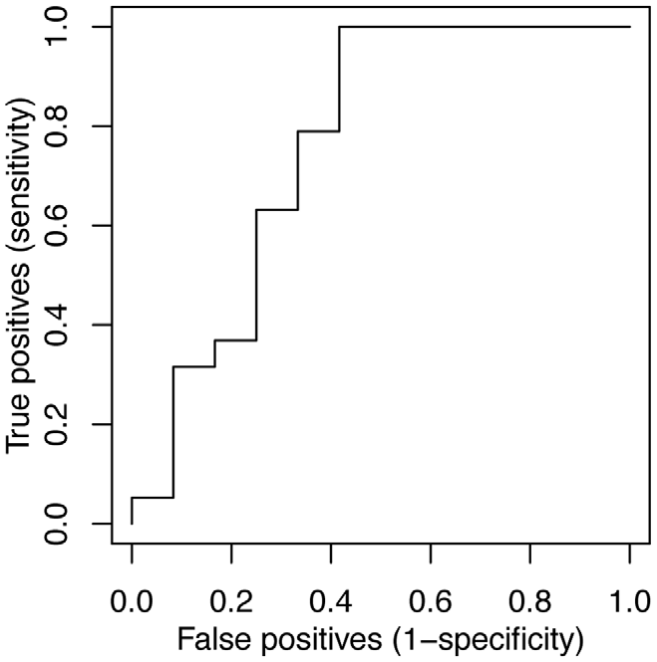
ROC analysis of the *BRCA2* signature in the validation set. Each tumor was given a score that was a weighted sum of the mean centered gene expression levels for each gene in the signature. The validation set contained 19 *BRCA2* and 12 *BRCAX* tumors. The AUC was 0.76.

**Figure 3:**
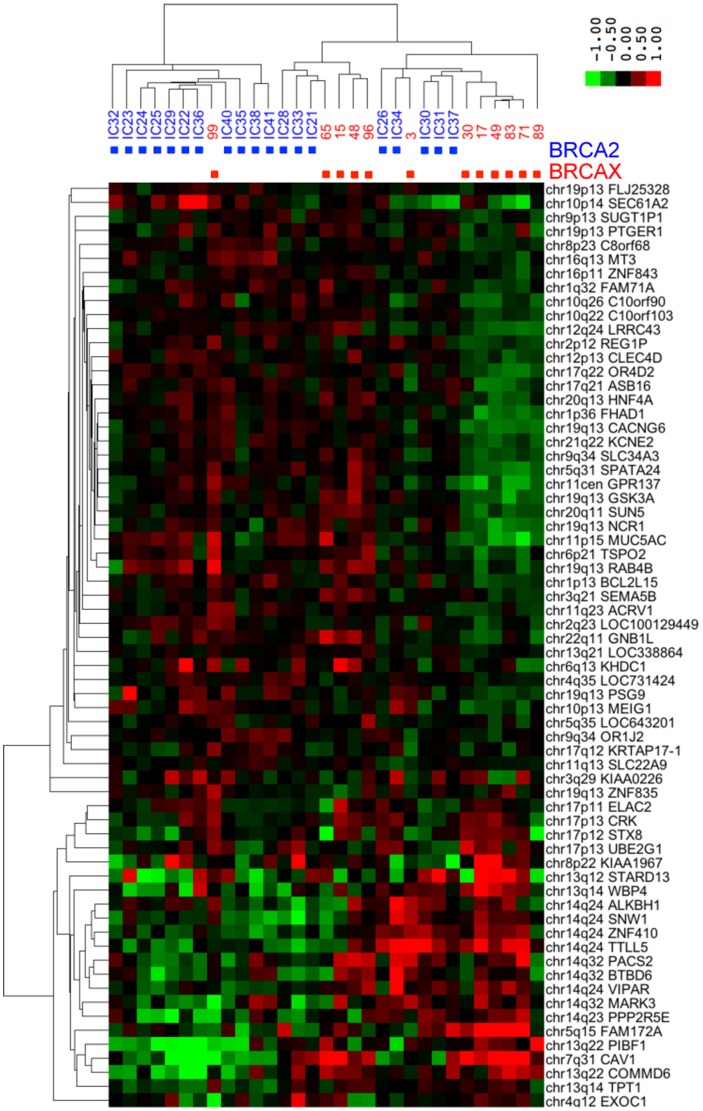
Unsupervised hierarchical clustering of the 66 *BRCA2* signature genes in the validation set. There are 19 *BRCA2-*mutant tumors and 12 *BRCAX* tumors. The lower left quadrant contains many genes on 13q and 14q that show reduced expression in *BRCA2* tumors.

We combined the differentially expressed genes in Table 2 to make a potential *BRCA2* gene expression signature. Receiver operating characteristic (ROC) analysis showed that the area under the curve (AUC) for classification of the training set was 1.0 with the *BRCA2* signature genes, indicating perfect classification of the tumors. This is not surprising given the small size of the dataset. To test for overfitting, we analyzed an independent validation set of 19 *BRCA2*-mutant tumors from the Curie Institute genetics clinic and 12 BRCAX from the Bergonie Cancer Institute. Given the rarity of the disease it is unfortunately difficult to avoid batch effects that might confound the result. Nevertheless, the AUC of the ROC curve was 0.76 in the validation set (Figure 2), indicating that the *BRCA2* signature was able to classify *BRCA2*-mutant tumors reasonably well. Hierarchical clustering confirmed that the *BRCA2* signature genes were differentially expressed in the validation set (Figure 3). While this suggests that the *BRCA2* signature has discriminant value in our tumors and in the validation set from the Curie Institute we note that this is not generally the case because the signature does not identify *BRCA2*-mutant tumors in some published datasets. For example, the AUC in the Waddell dataset [19] was 0.64, perhaps because of differences in the technology or in the populations studied. We conclude that the *BRCA2* signature may have discriminant value in tumors processed according to our protocol.

### Gene Set Enrichment Analysis (GSEA) Reveals the Mechanism Behind the *BRCA2* Signature

The striking feature of the heatmap in Figure 1 is the cluster of 22 genes showing reduced expression in *BRCA2*-mutant tumors. These genes show a correlation of 0.90 in the heatmap. To exclude fortuitous hybridization as an explanation for this strong clustering we verified that the probe sequences were different and that they were labeled by Affymetrix as valid, non-cross-hybridizing probes for the indicated genes. Fourteen of the 22 *BRCA2* signature genes showing reduced expression are from chromosomes 13 and 14. To determine whether this was due to chance, we ranked the dataset by moderated t statistic (*BRCA2* vs control), then performed GSEA with gene sets derived from individual chromosomal bands. The bands most frequently lost are shown in Table 3. The enrichment for bands on 13q and 14q was highly significant (p<0.001 for the family-wise error rate, the most stringent criterion in the Broad Institute implementation of GSEA). The most likely explanation for underexpressed genes to be derived from specific chromosomal bands is deletion of those bands in the corresponding tumors.

**Table 3:** GSEA for loss of chromosomal bands.

Band	Genes	ES	NES
13q14	67	−0.63	−2.75
14q31	22	−0.81	−2.71
13q13	22	−0.74	−2.45
14q24	77	−0.54	−2.43
17p13	185	−0.44	−2.3
14q32	105	−0.48	−2.28
10q26	72	−0.51	−2.27
4p16	91	−0.49	−2.25

Footnote. The genes column shows the number of genes used to score the band. The nominal, FDR and FWER p-values were all <0.001. ES, enrichment score; NES normalized enrichment score.

### CGH and SNP Analysis of BRCA2-mutant Tumors

**Figure 4:**
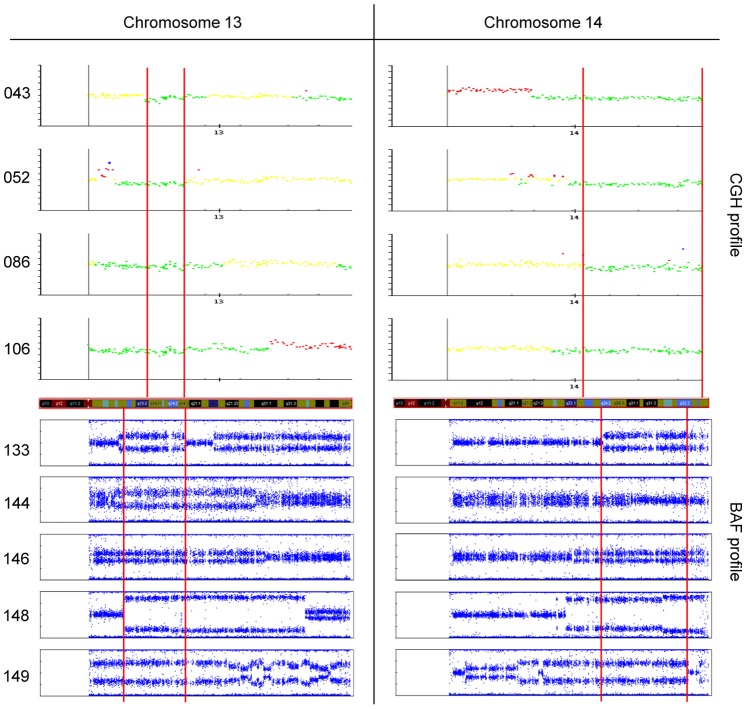
Genomic profiles in the training set. Upper panels: BAC-CGH profiles of *BRCA2*-mutant tumors showing gains in red, losses in green and modal copy number in yellow. Lower panels: BAF profiles of *BRCA2*-mutant tumors on Illumina SNP arrays. The boundaries of the common regions of deletion on chromosomes 13 and 14 are marked by vertical red lines.

**Figure 5:**
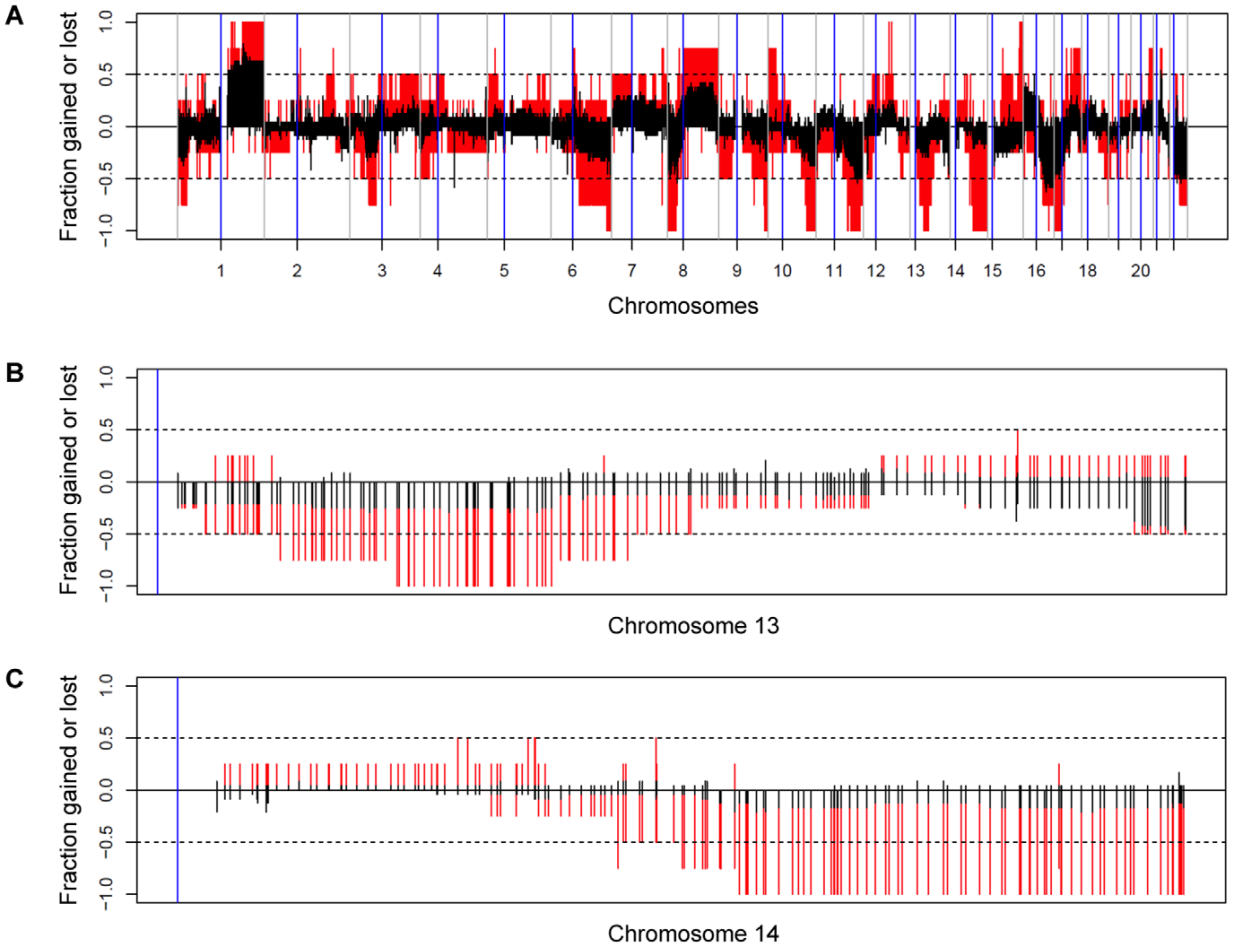
Cumulative rates of gain and loss for tumors analyzed by CGH (red, 4 *BRCA2*-mutant tumors; black, 24 *BRCAX* tumors). A, All chromosomes; B, Chromosome 13; C, Chromosome 14. Each vertical line in B & C corresponds to an individual BAC probe. When the red line reaches −1, all of the tumors showed loss for that probe.

To test directly for loss of the regions containing the *BRCA2* signature genes we measured DNA copy number on CGH and SNP chips. The resulting CGH and SNP profiles confirmed that the incriminated regions are indeed deleted in the *BRCA2*-mutant tumors (Figure 4). The common region of overlap of the deletions extends from 13q13.3 to 13q14.3 and from 14q24.2 to 14q32.2. The cumulative rates of gain and loss for the *BRCA2* and *BRCAX* tumors are shown in Figure 5. This shows that the long arms of both chromosomes 13 and 14 contain large regions that are preferentially deleted in the *BRCA2*-mutant tumors. We conclude that the *BRCA2* signature genes are differentially expressed because they are deleted in the *BRCA2* tumors.

### Identification of Deletions by FISH

**Figure 6:**
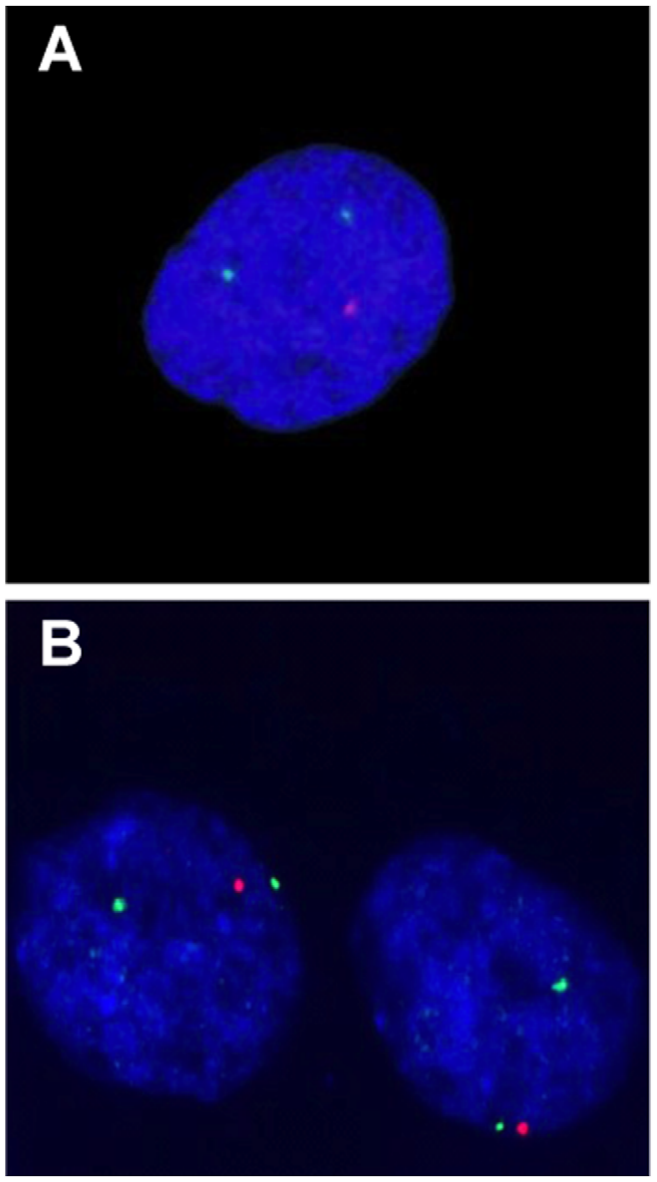
FISH with probes in the region of common deletion in a *BRCA2*-mutant tumor. A, chromosome 13; B, chromosome 14. Red: probe in the deleted region; Green, pericentromeric probe. Each nucleus contains two green spots and one red spot, indicating that the tumor is diploid for chromosomes 13 and 14 but has heterozygous deletions in the regions tested by the red probes.

If the signature works by detecting large deletions on chromosomes 13 and 14, it would be better to screen tumors in a clinical setting by FISH rather than by gene expression or CGH/SNP profiling. FISH is ideally suited to detecting small changes in copy number. To test whether it would be feasible to screen for *BRCA2*-mutant tumors in this way, we performed FISH with probes mapping to the regions commonly deleted on chromosomes 13 and 14 (Figure 6). We tested nine *BRCA2* tumors and nine control *BRCAX* tumors, of which five *BRCA2* and eight *BRCAX* were not previously characterized by CGH. The results are expressed as the percentage of nuclei with less than the modal number of spots for the centromeric probes or with a ploidy of one for both probes (Table 4). The tumors were scored as “loss” when the percentage was ≥50%, and “other” when it was <50%. Contingency tables for the chromosomes individually or for both chromosomes together are shown in Table 5. For both chromosomes scored together, the sensitivity and specificity for detection of *BRCA2*-mutant tumors were 78% and 89%, respectively. We conclude that FISH provides a simple technique to screen tumors for deletions on 13q and 14q that may be associated with *BRCA2* mutations.

**Table 4:** FISH with probes in the region of common deletion on chromosomes 13 and 14.

ID	BRCA status	chr 13	chr 14
52	BRCA2	84	89
86	BRCA2	90	86
106	BRCA2	100	87
133	BRCA2	93	89
A	BRCA2	84	83
B	BRCA2	100	0
C	BRCA2	87	7
D	BRCA2	100	62
E	BRCA2	100	73
16	BRCAX	0	0
F	BRCAX	0	2
G	BRCAX	0	0
H	BRCAX	100	100
I	BRCAX	4	0
J	BRCAX	0	0
K	BRCAX	2	0
L	BRCAX	7	3
M	BRCAX	10	0

Footnote. The table shows the percentage of nuclei with less than the modal ploidy or with ploidy = 1 for both the centromeric and the deletion probes. Tumours A-M were not characterized by CGH.

**Table 5:** Contingency table summarizing the FISH data for deletions on chromosomes 13 and 14.

Chr 13 and 14	Other	Loss
BRCA2	2	7
BRCAX	8	1
p = 0.015		
**Chr 13**	**Other**	**Loss**
BRCA2	0	9
BRCAX	8	1
p = 0.0004		
**Chr 14**	**Other**	**Loss**
BRCA2	2	7
BRCAX	8	1
p = 0.015		

Footnote. “Loss” refers to cases where ≥50% of nuclei had less than the modal ploidy or had ploidy = 1. “Other” refers to cases where the value was <50%. The p value is for a Fisher exact test. The values for “Chr13 and 14” refer to cases where both chromosomes were affected.

## Discussion

The main conclusion from our study is that deletions on chromosomes 13q and 14q are a common feature of *BRCA2*-mutant tumors. We initially set out to identify a gene expression signature that would distinguish these tumors from other tumors in patients presenting to our genetics clinics. Hierarchical clustering of the genes in the signature split the tumors into two groups in both the training and the validation sets, suggesting that the signature detects a signal that is useful for classification of the tumors. Given the GSEA and SNP/CGH results we strongly suspect that the reduced expression of the genes in the signature is caused by a reduction in the DNA copy number of the deleted regions. It is more difficult to detect deletion than amplification in gene expression data, because the former may further decrease a barely detectable signal whereas the latter can increase expression 100-fold. This probably explains why the genes in the signature are a minority of the genes in the deleted regions. Given the difficulty in measuring weakly expressed genes it is not surprising that previously reported *BRCA2* gene expression signatures did not highlight deletion of chromosomes 13 and 14 as a potential discriminating factor [19], [20]. In contrast, deletion of these regions was noted in several previous DNA copy number and SNP studies [7], [21]–[25]. In addition to published studies, we examined the GISTIC database (Tumorscape Release 1.6) [26] to determine whether loss of chromosomes 13 and 14 is a common event in breast cancer. Several regions are reported as harboring deletions on chromosome 13 (hg18 chr13∶44680312–57088104, 57088104–114059427, 18097312–46301361 and 50901262–114059427), as expected given the presence of *BRCA2* and *RB1* on 13 q. In contrast, GISTIC reports no regions as being deleted on chromosome 14 in breast cancer at above the background rate (q >0.25).

There are several possible explanations for selective deletion of specific genomic regions in *BRCA2* tumors. The commonly deleted region on chromosome 13 is distal to the *BRCA2* gene, but we can not altogether exclude that *BRCA2* itself may be a driver gene in some cases, for example if there were complex genomic rearrangements on 13 q. *BRCA2* was not part of the gene signature, probably because the Affymetrix probes for *BRCA2* are not sensitive enough (the measured level was close to background and showed minimal variation). The best reporters for copy number are housekeeping genes that lack feedback or exogenous regulation. By their nature these genes shed no light on the mechanism driving deletion. An alternative explanation is that loss of BRCA2 function generates repair intermediates or triggers checkpoint responses that are toxic in the presence of specific genes located in the deleted regions. Loss of these genes would allow the cell to resume division and form a tumor. This model predicts that the driver genes in the deleted regions should be DNA repair or checkpoint genes. *ALKBH1* could have this effect, but few other genes in the *BRCA2* signature are obvious candidates for these roles. Another possibility is that the deleted regions contain fragile sites that are more difficult to repair in the absence of BRCA2. Fragile sites are prone to replication fork collapse, a process that often leads to the formation of double strand breaks that require repair by homologous recombination. BRCA2 is required for loading of RAD51 to initiate homologous recombination [6] so increased breakage at fragile sites in the affected regions is certainly a possibility.

Screening for *BRCA2* mutations is widely performed in genetics laboratories to explain familial clustering of breast cancer. Our study design focused on patients referred to genetics clinics because this is the context in which the need to distinguish *BRCA2*-mutant from other tumors most commonly arises. Because of the size of the *BRCA2* gene it can take many months to identify mutations. This is rarely a problem in the context of genetic counseling because some interventions can be undertaken without knowledge of the mutation (for example, more frequent screening with imaging techniques) and others may even benefit from the delay by giving patients more time for reflection (for example, prophylactic mastectomy and oophorectomy). The same can not be said of medical treatment of established tumors, which must be delivered without delay. The advent of medical treatments specific for *BRCA2*-mutant tumors has created a need to identify these tumors on a more rapid time scale than has hitherto been considered necessary. In particular, *BRCA2* defects are synthetic lethal with inhibition of poly-ADP-ribose polymerase 1 (PARP1) [27], [28]. We note that the *BRCA2* group in the training set contains five *BRCAX* tumors which presumably either phenocopy *BRCA2* mutation or contain *BRCA2* mutations that evaded detection by sequencing. It would be interesting to know whether tumors that phenocopy *BRCA2* mutation are also sensitive to PARP inhibitors.

In the long term it is likely that diagnostic laboratories will routinely use next generation sequencing (NGS) to identify mutations in *BRCA2* and other relevant genes in the diagnostic biopsy when the patient initially presents with cancer. This is technically feasible but rarely performed outside major centers at present because of the cost and the complexity of the downstream bioinformatic analysis. To bridge the gap while waiting for NGS to become more widely available we propose to use FISH to screen breast tumors for deletions on 13q and 14q in order to identify tumors potentially associated with BRCA2. The technology for FISH is very well established for diagnosis of *ERBB2* amplification in sporadic breast tumors. It would require only a small modification of existing protocols to screen for loss of 13q and 14q in centers that already screen for *ERBB2* amplification by FISH. Patients whose tumors harbor deletions in those regions could then be screened by sequencing to identify either germline or somatic *BRCA2* mutations, followed by treatment with PARP inhibitors, if appropriate.

### Conclusion

We have shown that breast tumors arising in patients with germline *BRCA2* mutations have a higher frequency of deletions on 13q and 14q than is seen in other breast tumors. We propose that FISH for deletions on these chromosomes would be a rapid and technically feasible first step to enrich for tumors worth screening for *BRCA2* mutations. This would greatly facilitate the selection of patients for PARP inhibitor therapy.
